# Inspiratory muscle warm up improves 400 m performance in elite male runners

**DOI:** 10.1038/s41598-025-14797-0

**Published:** 2025-08-07

**Authors:** Yasemin Ari Yilmaz, Mehmet Ismail Tosun, Erkan Demirkan, Sema Can, Ali Özkan, Mustafa Arici, Mehmet Kutlu, Mert Ayranci, Milan Marković, İrem Eker Arici, Mustafa Onur Güneş, Tomasz Kowalski

**Affiliations:** 1 Department of Pulmonary Diseases, Faculty of Medicine, Hitit University, Çorum, Turkey; 2 Department of Physical Education and Sports, Faculty of Sport Sciences, Hitit University, Çorum, Turkey; 3 Department of Coaching Education, Faculty of Sport Sciences, Hitit University, Çorum, Turkey; 4 Department of Coaching Education, Faculty of Sport Sciences, Bozok University, Yozgat, Turkey; 5 Department of Recreation, Faculty of Sport Sciences, Hitit University, Çorum, Turkey; 6 Faculty of Sport and Physical Education, University of Priština-Kosovska Mitrovica, Leposavic, Serbia; 7 Turkish Athletics Federation, Ankara, Turkey; 8 Institute of Sport-National Research Institute, Warsaw, Poland

**Keywords:** Physiology, Respiration

## Abstract

This study sought to establish an optimal inspiratory warm-up (IWU) protocol for elite 400-meter athletes. It addresses the existing gap in sprint-specific research and provides practical applications for coaches and athletes aiming to optimize sprint running performance. Across four weeks, 13 elite male runners (age: 22.7 ± 2, personal best: 50.78 ± 0.65 s) performed four 400-meter time trials after different warm-up protocols in a randomized order. Assessments of various respiratory variables [maximal inspiratory pressure (MIP), maximal expiratory pressure (MEP), peak inspiratory flow rate (PIFR), inhaled volume (IV)] and selected physiological indices [heart rate (HR) and blood lactate (bLa)] were performed before and after the time trials. Analysis of variance with a significance level of *p* < 0.05 was applied. Incorporating IWU into a standard running warm-up resulted in a ~ 0.38-second faster 400-meter time trial in elite male sprinters (*p* < 0.001). The most effective IWU protocols, associated with the fastest run splits, consist of two sets of 30 dynamic inspiratory maneuvers performed with pressure threshold loading at 40% or 60% of MIP resistance. These protocols exhibited a positive influence on selected respiratory indices and significantly lowered post-run lactate concentrations compared to the athletic warm-up and sham protocol. IWU with 40% MIP resistance was associated with lower post-run HR compared to other protocols. IWU using pressure threshold loading at either 40% or 60% of MIP can be beneficial for elite male sprinters. While 40% MIP appears to support faster recovery, a higher intensity at 60% MIP shows greater effectiveness in enhancing 400-meter sprint performance. Future research should explore IWU applications across broader populations and conditions, including female athletes, various hypoxic environments, and temperature variations.

**Supplementary Information:**

The online version contains supplementary material available at 10.1038/s41598-025-14797-0.

## Introduction

 Track and field has been a fundamental component of the Olympic Games since ancient times, evolving into a highly specialized sport with three primary disciplines: running, jumping, and throwing. Among these, the 400-meter sprint remains a classic and popular event^1^. It is characterized as a speed-endurance event, necessitating a unique interplay between anaerobic and aerobic energy systems to sustain velocity throughout the race^2,3^. Unlike shorter sprints that rely almost exclusively on anaerobic energy production, the 400-meter race requires an optimal balance between anaerobic glycolysis (approximately 60–70%) and aerobic metabolism (30–40%)^4,5^. The duration of the race, typically ranging between 43 and 50 s at the elite level, results in substantial accumulation of bLa and hydrogen ions, leading to muscular acidosis and neuromuscular fatigue, particularly in the final 100-meters^6^. Such a physiological perspective distinguishes the 400-meter sprint and makes it one of the most metabolically challenging disciplines in track and field^7^. Quercetiani^8^ aptly described it as a “killer event” due to the extreme physical exertion required to maintain maximal velocity beyond the body’s natural capacity.

Given these physiological demands, researchers have extensively explored methods to optimize training, biomechanics, and psychological strategies to enhance 400-meter sprint performance^9–11^. However, despite these advancements, achieving even marginal improvements remains a critical objective in elite sprinting, where medal rankings are often decided by fractions of a second. This was evident in the 2024 Paris Olympic Games, where the time difference between the gold and fourth-place finishers in the men’s 400-meter final was a mere 0.38 s, while the gap between the bronze and fourth-place runners was only 0.04 seconds^12^. Such results highlight the importance of even the smallest physiological and biomechanical optimizations in determining race outcomes. One emerging area of interest in sprint performance optimization is respiratory muscle function, particularly the role of inspiratory muscles in sustaining and repeating high-intensity efforts^13–15^. During intense exercise, the increased ventilatory demand places a significant strain on the diaphragm and accessory respiratory muscles, potentially inducing inspiratory muscle fatigue and triggering the respiratory metaboreflex. This is a physiological response that redistributes blood flow away from locomotor muscles toward the respiratory muscles^16^. The respiratory metaboreflex reduces oxygen delivery to working limb muscles, accelerating peripheral fatigue and consequently impairing performance^17^. IWU may be an effective strategy to mitigate inspiratory muscle fatigue, enhance ventilatory efficiency, and delay fatigue onset^18^. However, most research on IWU has focused on endurance events, with limited literature examining its effects on sprint or prolonged sprint disciplines^19–21^. While mechanistic evidence suggests that IWU could be beneficial in events lasting less than one minute, such as the 400-meter sprint, studies on highly trained sprinters remain scarce, and no standardized warm-up protocol has been established^22^. Furthermore, existing research presents inconclusive findings regarding the effectiveness of different inspiratory warm-up protocols, particularly concerning variations in loading intensity and duration^23^. While IMW at 60%, 50%, and 40% of MIP has generally been reported to yield positive performance effects, IMW conducted at 15% MIP appears to be ineffective.

IWU derives from methods, techniques, and protocols established for respiratory muscle training (RMT). RMT is considered an ergogenic aid in sports performance, demonstrating benefits across multiple disciplines, including sprinting, endurance sports, and team-based activities^24,25^. IWU has been proven to enhance subsequent athletic performance by improving respiratory muscle strength, reducing perceived exertion, and optimizing oxygen kinetics^21,26,27^. However, the effectiveness of different IWU loads (low, moderate, or high resistance) remains unclear, as existing studies have reported inconclusive results^6,23^. The majority of IWU studies have utilized low-to-moderate inspiratory loads (5–40% of MIP), with 40% MIP remaining the most commonly tested resistance^22,28–31^. Fewer studies have examined the effects of higher inspiratory loads (50–80% MIP), which may provide greater respiratory muscle activation but also present a risk of inducing pre-fatigue, potentially attenuating performance benefits^6,23,32^. Moreover, studies in different populations report various outcomes^6,26,33^indicating that the optimal IWU protocol may depend on specific modality and training status. Since relevant research is lacking, the determination of the optimal IWU for elite 400-meter runners remains an unresolved question. Therefore, this study aimed to investigate the acute effects of different inspiratory warm-up protocols on 400-meter time-trial performance in elite sprinters. Moreover, various respiratory variables, bLa and HR were measured, as they are relevant for repeated performance, which frequently occurs during track and field competition^34,35^.

We hypothesized that higher inspiratory warm-up loads (60% MIP) would result in greater improvements in respiratory variables compared to lower loads (40% MIP and 15% MIP – SHAM protocol). We speculated that IWU with 60% MIP may simultaneously introduce additional fatigue, potentially offsetting performance gains and compromising sport-specific performance. Running performance, various respiratory indices, bLa, and HR kinetics were controlled. Overall, the study sought to establish an optimal IWU protocol for elite 400-meter athletes, addressing the existing gap in sprint-specific research. The findings are intended to inform coaches, practitioners, and athletes seeking to optimize sprint running performance through the application of IWU in high-performance settings.

## Methods

The study was conducted with the approval of the Hitit University Faculty of Medicine Research Ethics Committee and adhered to the principles outlined in the Declaration of Helsinki at all stages (Approval Number: 2024 − 148, 18/12/2024). Written informed consent was obtained from all participants. The trial was retrospectively registered on 20/03/2025 (NCT06886503), as participant recruitment and data collection had commenced prior to registration due to an initial administrative oversight. CONSORT reporting guidelines were applied where applicable.

### Participants

Thirteen male 400-meter sprinters were included in the final analysis. The participants’ best recorded times ranged between 49.71 s and 51.74 s (mean: 50.78 ± 0.65 s). The required sample size was determined with G*Power software (version 3.1.9.6; Dusseldorf, Germany), with a significance level of α = 0.05, statistical power (1 − β) = 0.95, and an effect size of ƒ = 0.5 (ANOVA with repeated measures, within interaction, 4 measurements), and totaled 10 participants. Large effect size was assumed based on the recent study from Marostegan et al. (2022), who investigated influence of IWU on 30 s all-out run 5. Initially, 14 athletes were recruited with convenience sampling by direct contact with coaches and athletes. One athlete dropped out during the study, did not complete all the required measurements, and was not included in further analyses(per-protocol approach). All the participants were highly-trained athletes specializing in 400-meter sprint competitions, training six days per week. All the participants were healthy, medically cleared to take part in competitive track and field, had previous competition experience in 400-meter track events at the national level, had no history of respiratory diseases, were non-smokers, and had not experienced any sports injuries in the past five months. The exclusion criteria were: using any ongoing medication, and any acute or chronic illness. The participants’ baseline characteristics, collected with standard protocols, are presented in Table 1.

**Table 1:** Baseline participants’ characteristics. Values are mean ± standard deviation.

Variable	Participants (*n* = 13)
Age [years]	22.7 ± 2.0
Body height [cm]	184.2 ± 2.2
Body mass [kg]	73.0 ± 2.3
Personal best for 400 m [s]	50.78 ± 0.65

### Experimental design

**Fig. 1:**
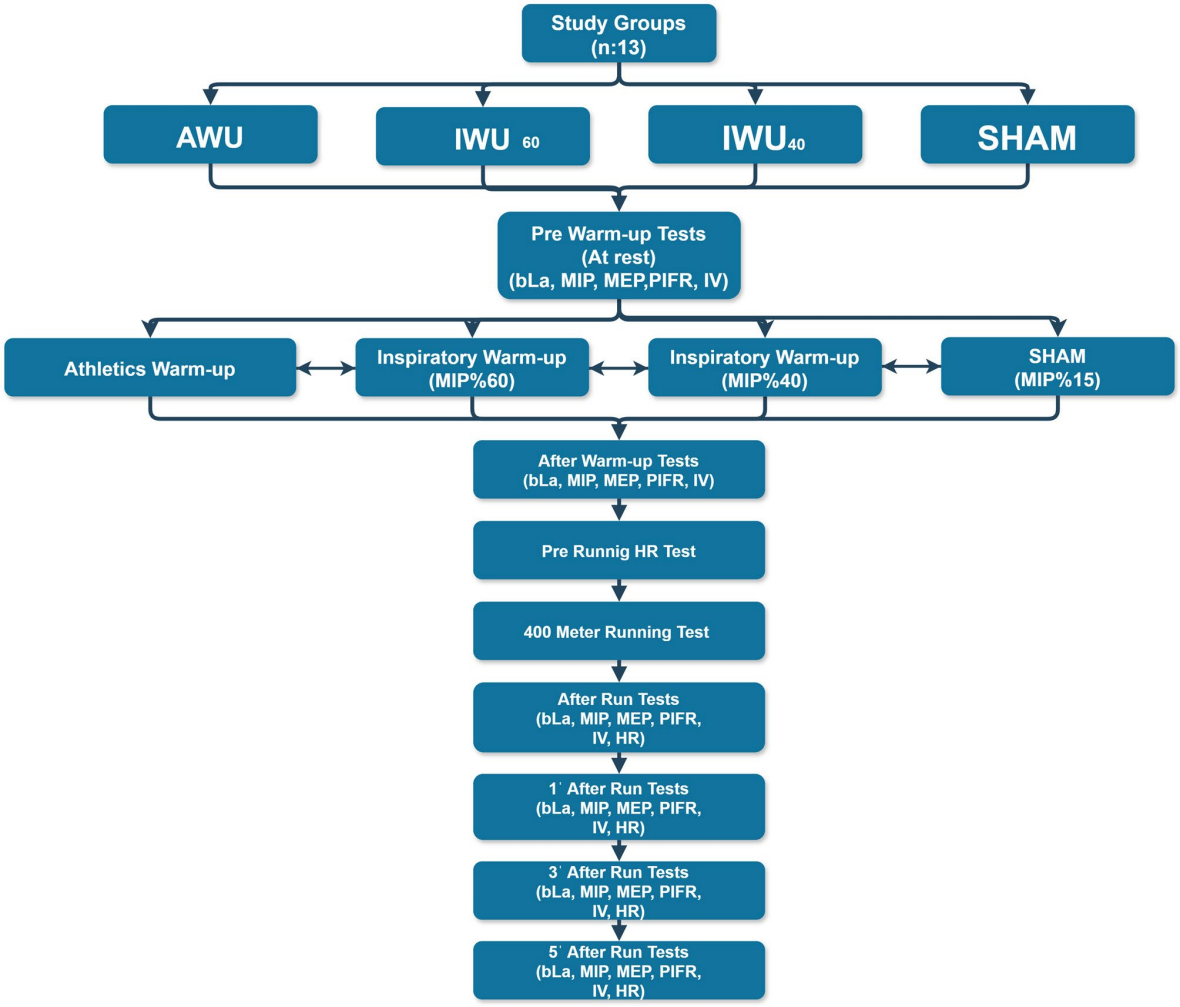
Flow chart.

 A single-blind, randomized-crossover design was employed in this study. Two days before the study commenced, all participants were provided with detailed information about the warm-up protocols and tests to be conducted. Participants were brought to the standardized 400 m track for measurements a total of four times, with one-week intervals, beginning two days after the initial familiarization and information session, and always conducted on the same day and time of the week (Friday, 2:00–3:00 PM). They were required not to perform any strenuous training and avoid long travels during the 24 h before the testing sessions. They were told to abstain from food intake for 3 h before the protocol and avoid alcohol, caffeine and ergogenic sport supplements for at least 24 h prior to testing to ensure controlled conditions. Additionally, participants reported their compliance with these instructions prior to each testing session. The participants underwent four different warm-up protocols in a randomized order, including an athletic warm-up (AWU) alone and AWU combined with three different IWU intensities (60% MIP, 40% MIP, and 15% MIP as the SHAM protocol).The participants were blinded to the applied IWU resistance. All warm-up protocols were administered one week apart, on the same day of the microcycle and at the same time, under relevant supervision. Physiological responses were assessed at predefined time points during each warm-up protocol session. Accordingly, respiratory function tests and bLa assessments were performed at the following time points: pre-warm-up, post-warm-up (pre-run), immediately after the 400 m time trial, and at 1, 3, and 5 min after the run (Fig. 1)^6,26^. Moreover, HR was recorded before the run, immediately after the 400-meter sprint, and at 1, 3, and 5 min after the run. All tests were performed by healthcare professionals (MD) and sports science researchers (PhD) with expertise in the respective measurements. To minimize assessor-related variability across all warm-up protocols, every measurement was carried out by the same researchers. The testing sessions commenced on 20/12/2024 and sessions were completed on 30/01/2025.

### Warm-up protocols

#### Athletic warm-up protocol

The athletic warm-up (AWU) consisted of 15 min of low-intensity running (rate of perceived effort 2–4 on 1–10 Borg Scale), followed by dynamic stretching exercises targeting the upper and lower extremities. In the final phase, running drills were performed, including three progressively increasing sprint efforts over a distance of approximately 30 m. Each sprint effort was separated by a rest interval of 2 min to ensure adequate recovery, and the timing of both sprint efforts and rest intervals was standardized using a stopwatch.

#### Inspiratory muscle warm-up protocols

In addition to the AWU protocol, participants performed IWU using the POWERbreathe Plus Blue Medium Resistance (POWERbreathe International Ltd., Warwickshire, UK) device at varying intensities. The IWU protocol was implemented at three different intensity levels: IWU_60_, IWU_40_, and SHAM. In the IWU_60_ protocol, IWU was performed at 60% of MIP, while in IWU_40_ and SHAM conditions, the intensity was set at 40% and 15% of MIP, respectively, with the latter serving as the SHAM protocol. Each protocol consisted of two sets of 30 repetitions, with a 90-second rest interval between sets^6,26^.

### Measurements

#### Respiratory variables

MIP represents the highest negative intrathoracic pressure generated during inspiration, while MEP refers to the highest positive intrathoracic pressure produced by expiratory muscles under static conditions. These physiological parameters are commonly used for the objective assessment of respiratory muscle strength^36,37^. For measurements, the Micro Medical/CareFusion MicroRPM (Micro Medical/CareFusion, Kent, UK) device, a validated assessment tool with real-time monitoring capability, was used^38^. To prevent air leaks, participants’ noses were occluded using a nasal clip during testing. For MEP measurement, participants were instructed to inhale maximally up to total lung capacity and then perform a forceful expiration through the mouthpiece. For MIP measurement, participants were instructed to exhale fully to residual volume, immediately followed by a maximal inspiratory effort. Each test was repeated at least three times, and testing was concluded when the difference between repeated measurements did not exceed 20%. The highest value was recorded as the final result. Participants were encouraged to exert maximum effort during all tests. All measurements were performed with participants in a seated position, following standardized guidelines^39^.

PIFR is a measurement used to assess respiratory muscle strength and airway resistance by measuring the maximum airflow rate (L/min) achieved during inspiration^40^. IV measures the total volume of air (L) inhaled in a single breath, serving as an indicator of respiratory muscle capacity and pulmonary ventilation efficiency^41^. A portable POWERbreathe K5 device (POWERbreathe International Ltd., Warwickshire, UK) was used for both assessments^42^. Testing was conducted with participants seated, their noses occluded with a nose clip^43^. Participants were instructed to exhale fully to residual volume, followed by a forceful maximal inspiration lasting at least one second. Each test was repeated three times, and the highest recorded value was used for analysis. Measurements of PIFR and IV were obtained simultaneously using the device’s single-breath test mode^26^.

#### Physiological indices

bLa was measured using a portable and validated analysis device, the Lactate Scout Sport (EKF Diagnostics, Barleben, Germany). All measurements were conducted according to standardized protocols to ensure accuracy and reliability. Capillary blood sampling was performed from the fingertip. Before sample collection, the skin was first cleansed and sweat was removed using an alcohol-based antiseptic, followed by rinsing with water for additional cleaning. A single-use lancet was then used to puncture the fingertip, and the first drop of blood was discarded. The subsequent drop was applied directly to the device’s test strip for analysis, and the blood sample was analyzed within a few seconds^44^.

A validated HR sensor (H10, Polar, Kempele, Finland) was used to measure HR, ensuring reliability and precision^45^. HR data were continuously monitored and recorded in real time using the Polar Flow mobile application, which synchronizes wirelessly via Bluetooth with the sensor to provide instantaneous feedback on cardiac activity. HR values were recorded as instantaneous readings at predefined measurement points during the testing protocol.

#### Determination of sprint time

After completing the warm-up protocol, participants performed a 400-meter sprint test on a standard 400-meter outdoor tartan track (lane 4) using their personal spiked running shoes previously used in competitions. The test was conducted using starting blocks, and at the beginning of each trial, a researcher with official sprint start officiating experience in track and field competitions gave the “Set” command. The sprint time was initiated automatically with the sound of a starting pistol (Gill Athletics., E49710 – Gill Halo, Champaign, USA) connected to an optical sensor. Participants ran with maximum effort towards the finish line, where the time was stopped using optical sensors (Dolunay Electronics Inc., DK-386, Ankara, Türkiye). Performance was recorded automatically. Participants were instructed to perform as if they were in a competitive race, but no pacing or race strategy guidance was provided. Due to the frequency of post-run measurements, each test was conducted individually to ensure accurate data collection.

### Statistical analysis

The normality of the distribution was evaluated with the Shapiro-Wilk test and visual assessment. The basic results are reported as mean and standard deviation. The statistical effects for running performance were evaluated by regular analysis of variance. The effects for time and the interaction between time and warm-up protocol for all other variables were analyzed using analysis of variance for repeated measures. Mauchly’s test of sphericity and Greenhouse-Geisser correction were used to identify and correct for the violation of sphericity. To account for multiple testing, the post-hoc Holm correction was applied, ensuring a stringent control of type I error. Effect sizes were calculated using partial eta squared (ηp²) and omega squared (ω²). The following values have been suggested for effect sizes: small (0.01), medium (0.06), and large (0.14)^46,47^. A significance level of *p* < 0.05 was applied. All statistical analyses were performed using the JASP Team statistical package JASP (Amsterdam, Netherlands, version 0.17.2).

## Results

Both warm-up and 400 m time trial were associated with significant changes for all the measured variables (*p* < 0.001, effect sizes from 0.446 to 0.999 for ηp² and from 0.205 to 0.997 ω²). Most importantly, the 400 m time trial induced significant disturbance in homeostasis, represented by deterioration of respiratory function and increases in HR and bLa. The detailed values are presented in Table 2.

**Table 2:** Changes of all the measured respiratory and physiological indices in time. Values are mean and standard deviation. MEP: maximum expiratory pressure; MIP: maximum inspiratory pressure; PIFR: peak inspiratory flow rate; IV: inhaled volume; HR: heart rate; bLa: blood lactate.

Measurement/ Variable	MIP	MEP	PIFR	IV	HR	bLa
p-value	< 0.001	< 0.001	< 0.001	< 0.001	< 0.001	< 0.001
Effect size [ηp²/ω²]	0.928/0.535	0.802/0.510	0.859/0.311	0.446/0.205	0.993/0.983	0.999/0.997
At rest	139.6 ± 6.1	174.3 ± 13.2	6.20 ± 0.5	3.87 ± 0.23	-	0.9 ± 0.2
After warm-up	145.8 ± 7.9	179.1 ± 13.7	6.31 ± 0.5	3.93 ± 0.21	103.3 ± 3.3	3.9 ± 0.3
Immediately after run	125.9 ± 9.1	151.6 ± 9.4	5.45 ± 0.5	3.53 ± 0.49	183.4 ± 4.0	17.9 ± 0.4
1ˈ after run	128.9 ± 9.9	153.1 ± 9.7	5.50 ± 0.5	3.61 ± 0.23	156.9 ± 5.1	17.8 ± 0.6
3ˈ after run	132.1 ± 9.8	156.4 ± 10.0	5.61 ± 0.5	3.68 ± 0.25	112.0 ± 5.8	17.8 ± 0.8
5ˈ after run	133.5 ± 10.0	158.9 ± 12.4	5.68 ± 0.5	3.71 ± 0.26	103.8 ± 4.9	14.9 ± 0.8

A significant effect of the applied protocol was observed for running performance (*p* < 0.001, ηp²= 0.639, ω²= 0.061). The post-hoc test exhibited no significant differences between AWU and SHAM (*p* = 0.501). However, there were significant differences for all the other comparisons (*p* = 0.019 for IWU60 vs. IWU40, and *p* < 0.001 for all the remaining pairs). The detailed values are presented in Table 3.

**Table 3:** Different warm-up protocols and subsequent running performance. Values are mean and standard deviation. AWU: athletic warm-up; IWU_60_: athletic warm-up and subsequent inspiratory muscle warm-up at resistance corresponding to 60% of maximum inspiratory pressure; IWU_40_: athletic warm-up and subsequent inspiratory muscle warm-up at resistance corresponding to 40% of maximum inspiratory pressure; SHAM: athletic warm-up and subsequent inspiratory muscle warm-up at resistance corresponding to 15% of maximum inspiratory pressure.

Measurement/Variable	AWU	IWU_60_	IWU_40_	SHAM
400 m time [s]	50.811 ± 0.65	50.425 ± 0.52	50.576 ± 0.58	50.774 ± 0.64
Change compared to AWU [% difference]	0	−0.76	−0.44	−0.073

**Fig. 2:**
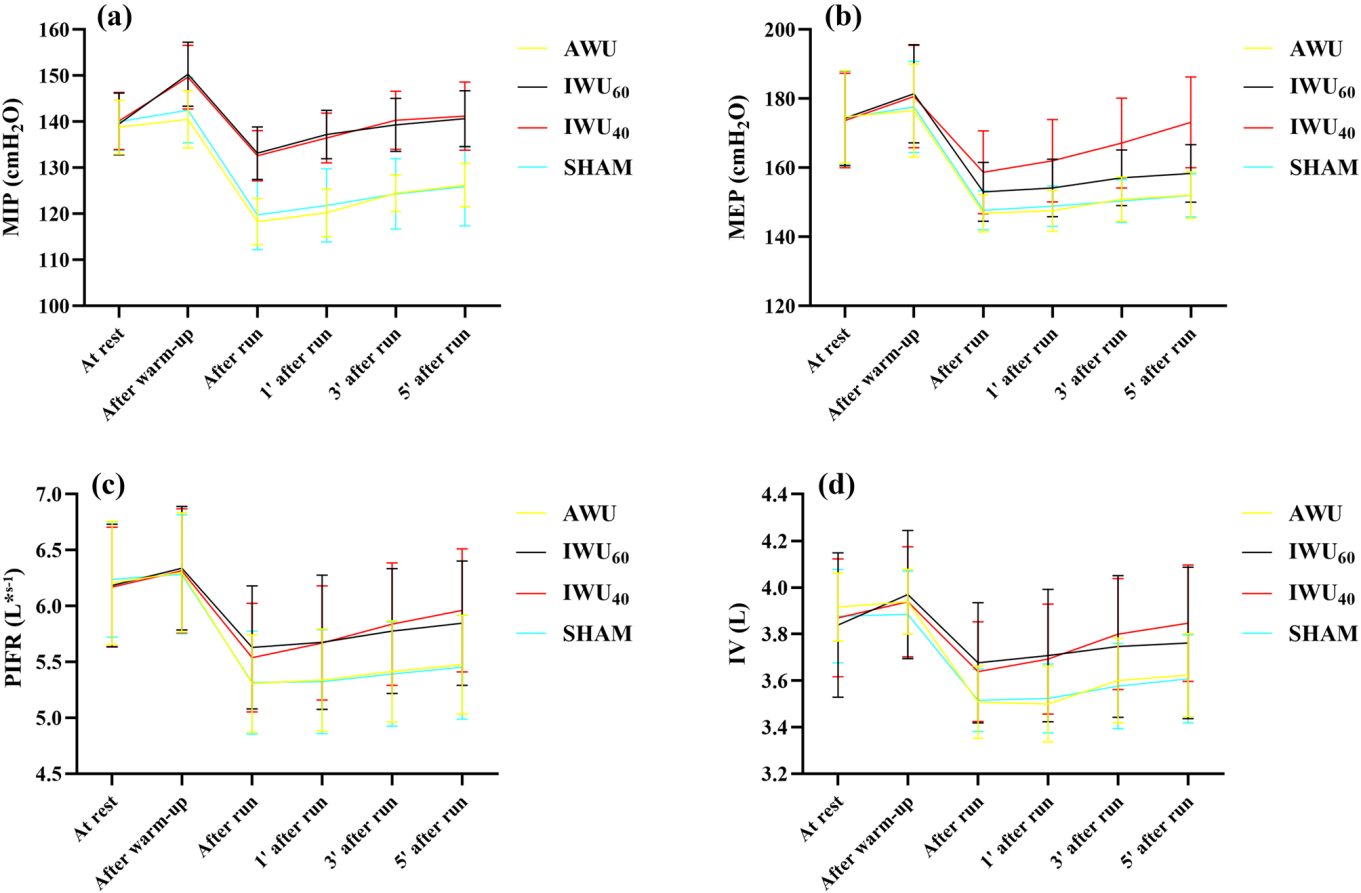
Changes in respiratory variables at different time points for different warm-up protocols. **(A-D)** Maximal inspiratory pressure, maximal expiratory pressure, peak inspiratory flow rate, and inhaled volume across different warm-up protocols and measurement times. AWU: athletic warm-up; IWU_60_: athletic warm-up and subsequent inspiratory muscle warm-up at resistance corresponding to 60% of maximum inspiratory pressure; IWU_40_: athletic warm-up and subsequent inspiratory muscle warm-up at resistance corresponding to 40% of maximum inspiratory pressure; SHAM: athletic warm-up and subsequent inspiratory muscle warm-up at resistance corresponding to 15% of maximum inspiratory pressure; MIP: maximum inspiratory pressure; cmH_2_O: the pressure exerted by a 1-centimeter column of water; MEP: maximum expiratory pressure; PIFR: peak inspiratory flow rate; L·s⁻¹: breathing airflow in liters per second; IV: inhaled volume; At rest: measurement taken while the athlete is in a resting state before the warm-up protocol; After warm-up: measurement taken immediately after the warm-up protocol and just before the 400-meter run; After run: measurement taken immediately after the 400-meter run; 1ˈ after run: measurement taken 1 min after the 400-meter run; 3ʹ after run: measurement taken 3 min after the 400-meter run; 5ˈ after run: measurement taken 5 min after the 400-meter run.

**Fig. 3:**
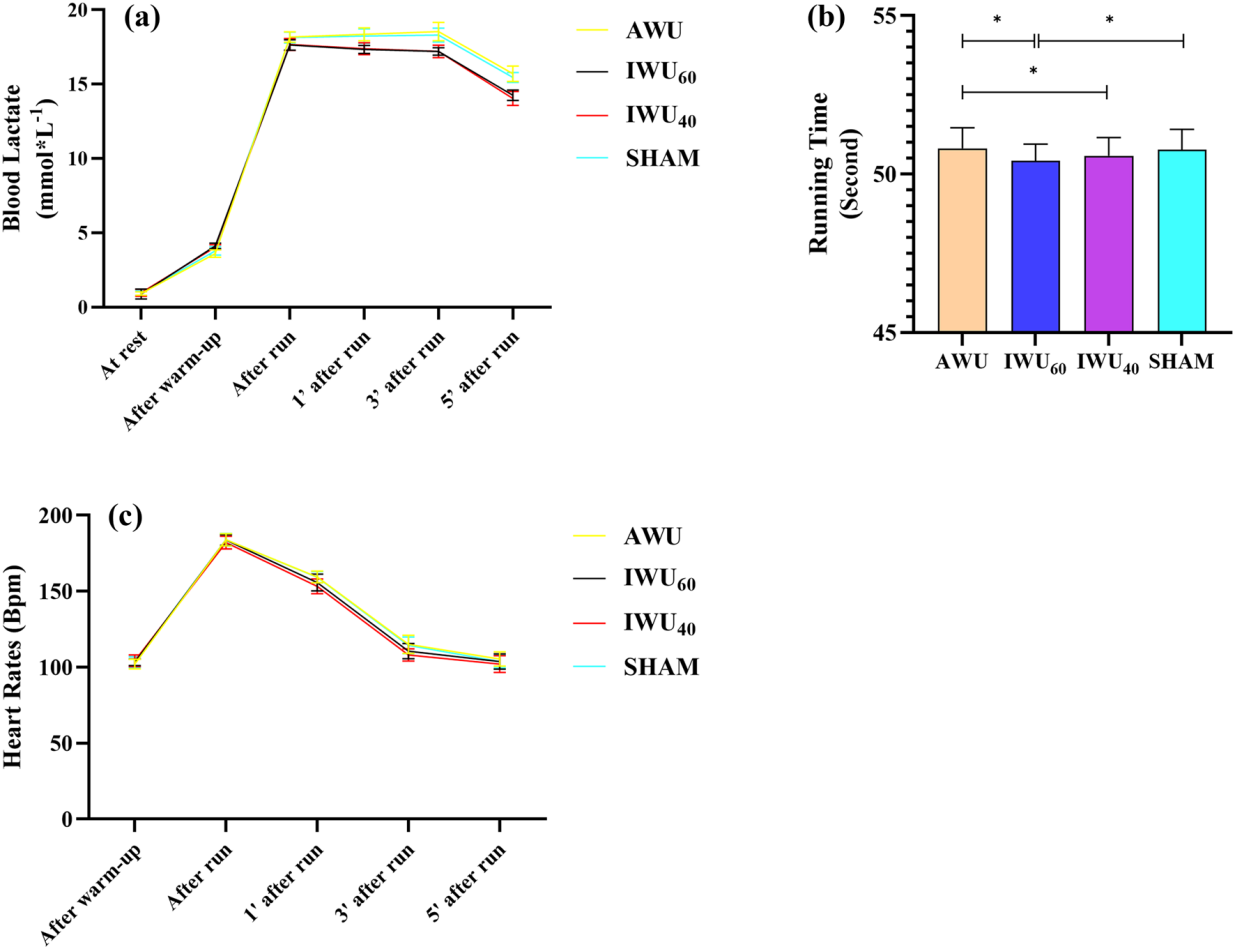
Changes in physiological indices at different time points for different warm-up protocols. **(A**,** C)** Blood lactate and heart rate responses across different warm-up protocols and measurement times. **(B)** 400-meter running time-trial across different warm-up protocols. AWU: athletic warm-up; IWU_60_: athletic warm-up and subsequent inspiratory muscle warm-up at resistance corresponding to 60% of maximum inspiratory pressure; IWU_40_: athletic warm-up and subsequent inspiratory muscle warm-up at resistance corresponding to 40% of maximum inspiratory pressure; SHAM: athletic warm-up and subsequent inspiratory muscle warm-up at resistance corresponding to 15% of maximum inspiratory pressure; mmol^*^L^−1^: concentration of lactate in blood, expressed in millimoles per liter.

Finally, there were significant differences for time*protocol changes for all the measured variables except for IV (*p* < 0.05). Therefore, post-hoc tests were applied to allow for pairwise comparisons between the groups while controlling for the increased risk of type I errors. A summary of the findings is presented in Table 4; Figs. 2 and 3. The detailed tables are included in Supplementary Materials (Table S1, Table S2, Table S3) 1. In particular, the post-hoc tests showed that:

1. There were no differences at rest for all the variables. However, in the IWU_60_, IWU_40_ and SHAM groups, MIP was significantly higher after the warm-up compared to AWU. Moreover, bLa was significantly higher after the warm-up in IWU_60_ compared to all the other protocols.
2. The post-run values for MIP were significantly lower in AWU and SHAM compared to IWU_60_ and IWU_40_.
3. The post-run values for bLa were significantly higher in AWU and SHAM compared to IWU_60_ and IWU_40_.
4. MIP was significantly lower in AWU and SHAM compared to IWU_60_ and IWU_40_ at 1, 3, and 5 min after the run. Mixed findings were observed for MEP, in favor of IWU_40_. Mixed findings were observed for HR, in favor of IWU_60_ and IWU_40_.
5. bLa was significantly higher in AWU and SHAM compared to IWU_60_ and IWU_40_ at 1, 3, and 5 min after the run.

**Table 4:** Differences for time*protocol analysis of variance in measured physiological indices. MIP: maximum inspiratory pressure; MEP: maximum expiratory pressure; PIFR: peak inspiratory flow rate, IV: inhaled volume; HR: heart rate; bLa: blood lactate.

Measurement/Variable	MIP	MEP	PIFR	IV	HR	bLa
p-value	< 0.001	0.002	< 0.001	0.064	< 0.001	< 0.001
Effect size [ηp²/ω²]	0.696/0.144	0.261/0.047	0.326/0.026	0.129/0.016	0.195/0.038	0.638/0.293

## Discussion

### Summary of main findings

The study aimed to establish an optimal IWU protocol for elite 400-meter athletes by examining the effects of different loads on respiratory indices, HR, bLa concentration, and 400-meter performance in elite runners. To the best of our knowledge, this is the first study to compare the effects of AWU, IWU_40_, IWU_60_, and SHAM protocols on wide array of physiological and performance variables in elite 400-meter sprinters during specific sprint effort. IWU_60_ and IWU_40_ were associated with the greatest improvement in running time, outperforming AWU by 0.76% (0.38 s) and 0.44% (0.23 s), respectively. Our findings revealed that warm-up protocols based on 60% and 40% of MIP lead to significantly higher MIP values after the warm-up and running trial compared to AWU and SHAM protocols (5.03 to 14.13%). However, our study findings revealed no difference between IWU_60_ and IWU_40_.

The IWU_60_ protocol performed best regarding post-exercise bLa clearance, showing 2.86% lower levels compared to the AWU condition and 2.70% lower levels compared to the SHAM protocol. This difference became more pronounced at the 5th minute of recovery, where IWU_60_ resulted in 9.22% lower bLa levels than AWU and 6.84% lower than SHAM. Similarly, the IWU_40_ protocol yielded improved bLa clearance, with post-exercise bLa levels 2.75% lower than AWU and 2.59% lower than SHAM. At the fifth minute of recovery, these reductions reached 10.69% and 9.19%, respectively, highlighting the effectiveness of IWU40 in the later stages of recovery.

### Performance effects and recovery indicators

An interesting finding was the time-dependent variation between the IWU_60_ and IWU_40_ protocols. Immediately after exercise, IWU_60_ produced a 0.02 mmol (0.11%) lower bLa level than IWU_40_. However, this difference was reversed by the 5th minute of recovery, with IWU_40_ showing a 1.61% (0.23 mmol) lower bLa level than IWU_60_, indicating superior performance at this recovery stage. Although this difference did not reach statistical significance (*p* > 0.05), the trend suggests that IWU_40_ may promote more efficient lactate clearance during recovery.

Regarding HR, another critical physiological parameter in assessing recovery, the most favorable results were observed with the IWU_40_ protocol. At 1 min after running measurement, IWU_40_ achieved a 3.9% lower HR compared to AWU and 3.76% lower than SHAM. It also outperformed IWU_60_, with a 1.62% lower HR. The IWU_60_ protocol, in turn, demonstrated a notable recovery effect by reducing HR by 2.37% and 2.22% compared to AWU and SHAM, respectively. These differences were also not statistically significant but indicate a trend toward improved autonomic recovery with IWU protocols.

### Comparison with existing literature

The results of our study align with recent findings from other authors that demonstrated the positive effects of IWU on respiratory function in healthy individuals. Arend et al.^21^ demonstrated that the 40% and 60% MIP warm-up protocols resulted in acute increases in MIP of approximately 5.2% and 4.8%, respectively, in a sample of ten healthy adult males. Demirkan et al.^24^ eported a significant acute increase in MIP after the IWU (40% of MIP) plus athletic warm-up, with a 17.3% improvement compared to the athletic-only and SHAM warm-up conditions (2.3% and 7.8%, respectively) in a group of fourteen adolescent male wrestlers. Lomax et al.45 found that IWU alone acutely increased MIP by approximately 11%, and also improved running distance during the Yo-Yo intermittent recovery test by 5–7% in twelve healthy county-standard and semi-professional male football players.Özdal^18^ stated that after the IWU, there was a significant elevation of 7.03% for MIP and 12.52% for IV, compared to the control group. In our study, we observed a smaller post-run MIP decline for IWU_60_ and IWU_40_ (−11.41 and − 11.39%, respectively), compared to AWU and SHAM (−15.81 and − 15.97%, respectively). Volianitis et al.^48^ reported that respiratory warm-up combined with a specific rowing warm-up significantly reduced inspiratory muscle fatigue to 4.2%, compared with 10.2% after a submaximal rowing warm-up and 11.1% after a specific rowing warm-up alone. Lin et al.^29^ noted that IWU (40% of MIP) improved inspiratory muscle function by 7.8% and 6.9% compared to the controls. Lomax & McConnell^30^ found that IWU enhanced the inspiratory pressure by 11–17%, irrespective of training status. In another study, Ohya et al.^49^ observed that MIP values were significantly higher under IWU conditions compared to SHAM conditions.

The acute improvement observed in MIP capacity may be explained by increased motor unit activation^50^enhanced neuromuscular facilitation, and the contribution of the post-activation potentiation mechanism^26,27^. In our study, PIFR and IV values were not significantly higher after IWU_60_ and IWU_40_ in comparison to AWU and SHAM (Fig. 2c-d). Our findings confirmed that the inclusion of IWU (60% of MIP or 40% of MIP) increases MIP after warm-up and subsequent high-intensity exercise.

### Physiological mechanisms

Furthermore, in this study, in comparing the decline of MEP after the IWU, it was observed that the smallest statistically significant decline was seen in IWU_40_ compared to other IWU protocols (Fig. 2b). In other words, MEP after the running remained higher in 40% of the inspiratory load for all the repeated measurements. Hence, IWU at 40% of MIP could positively affect the running performance by delaying respiratory muscle fatigue. The preservation of MEP following IWU, particularly at 40% of MIP, may play a critical role in sustaining expiratory muscle function during high-intensity efforts such as sprinting. Higher MEP contributes to more effective ventilation by facilitating CO₂ clearance and stabilizing intrathoracic pressure, which can support efficient gas exchange and delay the onset of respiratory muscle fatigue. This may reduce the perception of dyspnea during the final stages of sprinting, where respiratory and locomotor demands peak.

Moreover, respiratory muscle fatigue causes the metaboreflex, which restricts blood flow to the limbs by increasing sympathetic vasoconstrictor outflow, leading to locomotor muscle fatigue, which limits exercise performance^51^. Cheng et al.^22^ reported that oxygen saturation in the local working muscle was enhanced by the specific warm-up activity of 40% of MIP, delaying the activation of the respiratory muscle metaboreflex. Katayama et al.^52^ observed that inspiratory muscle exercise facilitated sympathetic control of blood redistribution to active limbs by the respiratory muscle-induced metaboreflex. Additionally, it has been suggested that a specific IWU in combination with a whole-body warm-up could reduce breathlessness, enhance dynamic respiratory function, and improve exercise tolerance in subsequent exhaustive intermittent runs^28^. Collectively, the attenuation of respiratory muscle fatigue and delayed onset of the metaboreflex may help preserve cardiac output distribution during intense exercise by preventing excessive sympathetic-mediated vasoconstriction in active musculature. This mechanism supports sustained oxygen delivery to working limbs, mitigates peripheral fatigue, and ultimately contributes to improved systemic performance under high metabolic demand.

### Practical implications for elite athletes

There were no differences between the IWU loads of 60% and 40% of MIP regarding the run performance (Fig. 3b). Although the difference in running performance between IWU_60_ and IWU_40_ was not statistically significant, IWU_60_ yielded approximately 0.15 s faster times. While this may seem marginal, such a difference could be meaningful in sprint events where outcomes are often determined by hundredths of a second. Nevertheless, given the similar performance outcomes and the potentially lower risk of fatigue with IWU_40_, this lower-intensity protocol may offer a more practical and efficient preparatory strategy for elite sprinters.

However, our findings indicated that the running velocity was non significantly higher after IWU_60_ compared to IWU_40_. In addition, the IWU loads of 60% and 40% of MIP significantly enhanced running performance compared to the other protocols (Fig. 2a). Based on the times of running performance in our study, it might be suggested that IWU of 60% combined with a specific warm-up could play an important role in reducing the time in 400-meter running performance. The observed performance enhancement may be linked to the IWU performed before running, which is proposed to improve the functional capacity of the respiratory muscles. This intervention is thought to enhance ventilatory efficiency, delay the onset of respiratory fatigue, optimize blood flow to the lower extremities, and support more effective utilization of anaerobic energy pathways^20,53^. Additionally, pre-activation of the inspiratory muscles may reduce the respiratory workload, facilitating greater oxygen delivery to the working skeletal muscles. This, in turn, may accelerate motor unit recruitment and contribute to the maintenance of stride frequency and length^6^. Improved regulation of intrathoracic pressure may further enhance postural stability and minimize energy loss, while an elevated ventilatory threshold could help sustain lactate buffering capacity. Collectively, these physiological adaptations are thought to contribute to the observed improvements in sprint performance^54^.

### Consistency and discrepancy with other studies

Most of the available literature, although regarding a similar but not exact context, is consistent with our findings. Wilson et al.^31^ reported significant benefits for swimming performance after using a swimming warm-up plus IWU at 40% MIP. The authors stated that the inspiratory muscle exercise combined with a standard swimming warm-up significantly improved swimming performance in elite swimmers. Özdal et al.^55^ stated that respiratory warm-up exercise significantly improved peak power and time to achieve peak power. Barnes & Ludge^21^ reported that IWU showed a positive effect (~ 21 s, 2.8%) on 3200 m running performance. Manchado-Gobatto et al.^56^ stated that the pre-activation of the inspiratory muscles at 40% of the maximal inspiratory pressure improved the running power and enhanced recovery. Avci et al.^57^ reported that drag-flick and shot hit performance in hockey was improved by using inspiratory muscle exercise. Lomax et al.^50^ noted that both RMT and IWU increased running distance separately; however, when the combination of both was performed, the covered distance was the greatest^50,58^. In another study, Cirino et al.^59^ observed an improvement in Ippon scores after performing IWU at 40% of MIP compared to 15% of MIP in a simulated judo setting. Unlike the results of other studies, Arend et al.^60^ found that performing an inspiratory muscle warm-up at 40% of MIP did not lead to any significant improvement in performance—measured by time or distance—during a submaximal rowing ergometer test at 90% of MIP in well-trained male rowers.

In another study, Faghy & Brown^61^ noted that athletic warm-up and IWU performed alone or together had no effect on high-intensity, short-duration performance. Merola et al.^32^ reported that high-load IWU (60% of MIP) combined with a specific judo warm-up in elite judo athletes did not lead to an improvement in judo performance. Johnson et al.^62^ stated that there was no performance enhancement in a 10-km cycling time trial, after performing a cycling warm-up combined with IWU. Another study finding revealed no ergogenic effect on performance when using IWU during a 3000 m time trial in elite speed skaters^63^. Ohya et al.^49^ noted that IWU improved inspiratory muscle function, but this improvement had no positive effect on high-intensity intermittent sprint cycling exercise in untrained healthy males. Soares de Araujo et al.^64^ reported that the IWU protocol at 40% of the MIP did not improve high-intensity tethered swimming. Such inconclusive findings may stem from different conditions of the studies, including subjects’ status, IWU loads, the environment, and other factors.

In our study, IWU was associated with a significantly higher bLa at the load of 60% and 40% of MIP compared to other warm-up protocols (Fig. 3a). Accordingly, IWU may cause an acute increase in bLa concentration. However, the 60% and 40% of MIP warm-up protocols caused a significantly larger decline in bLa after exercise, in the first, third, and fifth post-run minute. A study conducted by Cheng et al.^22^ demonstrated that the IWU of 40% of MIP led to greater protection against a decrease in muscle oxygen saturation in submaximal cycling exercise and the intermittent high-intensity sprint exercise. In a study supporting this result, Marostegan et al.^6^ found that 40% of the MIP improved tissue saturation index of the biceps brachii in the recovery phase, which could lead to greater provision of O_2_ for lactate elimination. Additionally, other studies showed that inspiratory resistance loading improved metabolic acidosis conditions after high-intensity interval sprints^5^ and subsequent supra-maximal exercises^65^. According to the findings, IWU can be a potential strategy to support lactate elimination, especially after high-intensity exercise. The reason for this in previous studies was that the diaphragm and respiratory muscles led to increased blood flow. Therefore, it could be caused by an increased use of bLa as an energy source^60^. Brown et al.^66^ reported that RMT of 6 weeks improved both lactate exchange and clearance. In agreement with these findings, the inspiratory resistance during recovery from intense exercise leads to increased oxygen uptake, reduced bLa, and changes in breathing pattern^29,67^. Furthermore, Chiappa et al.^67^ noted that inspiratory muscles could be net consumers of lactate during recovery from intense exercise. Additionally, the findings of HR changes in this study showed that 40% of MIP warm-up protocols significantly reduced HR (by 3.90–6.14%) in the first and third recovery times in comparison with AWU and SHAM protocols. Based on these findings, we suggest that IWU could support faster recovery by reducing the lactate concentration and HR, especially after high-intensity, short-duration exercises. Additionally, other studies^57,68–70^ have demonstrated that RMT showed a positive effect on reducing respiratory muscle fatigue and local muscle fatigue. However, contrary conclusions were reported in a few studies. Soares de Araujo et al.^64^ reported that IWU did not reduce bLa concentrations immediately after the tethered swimming test. Thurston et al.^19^ noted that the bLa did not change in association with IWU in healthy men who exercised recreationally. Finally, IWU may be associated with an additional training load even in trained athletes^71^. Therefore, it should be applied with caution in high-performance settings to avoid possible overreaching or overtraining.

## Conclusion

This study demonstrated that IWU based on pressure threshold loading at 40% and 60% of MIP, when combined with a standard running warm-up, can effectively enhance 400-meter sprint performance in elite male athletes. Both loading intensities resulted in notable improvements in inspiratory muscle strength and contributed to attenuated lactate accumulation during the recovery period, suggesting improved post-exercise metabolic efficiency. Although no significant differences were observed between the 60% and 40% MIP protocols in terms of MIP improvements, IWU_60_ elicited a slightly greater enhancement in running velocity, whereas IWU_40_ was more effective in preserving MEP and lowering post-exercise HR.

These findings point to a potential ergogenic role of submaximal IWU, particularly in reducing respiratory muscle fatigue and supporting faster recovery after high-intensity, short-duration efforts. Importantly, while the 60% MIP warm-up may be more suitable for performance enhancement in high-intensity intervals, the 40% protocol appears advantageous for recovery phases between efforts. Future studies should explore the applicability of these protocols in diverse populations, including female athletes, and under various environmental conditions such as hypoxia and temperature extremes.

### Strengths, limitations, and recommendations for further research

The primary strength of this study lies in the innovative approach, i.e. considering recent peer-reviewed findings to suggest and verify practical and feasible IWU protocols. Importantly, all procedures were performed with validated equipment, under the close supervision of skilled professionals, and every measurement across all protocols was conducted by the same assessors to minimize inter-rater variability. While the sample size may be relatively small, the study benefits from the participation of a distinctive group of highly trained sprinters. However, several limitations warrant consideration. Some points need to be made about the limitations of the present research. First, this study was conducted on males only, which limits the versatility of the findings. Next, the testing period spanned 22 days. Throughout this period, many variables associated with respiratory and sport-specific performance may have experienced minor fluctuations due to typical temporal variations, training adaptations, or even the repeated-bout effect. Moreover, different week-to-week environmental conditions (in particular temperature and humidity) may have also influenced the running performance. However, since the appropriate randomization was applied, these should not have influenced the robustness of the results. Future studies on IWU should encompass diverse populations and training environments, particularly focusing on differences between genders, as current research on female athletes remains scarce. Additionally, exploring the impact of various hypoxic conditions and temperatures on IWU effectiveness would provide valuable insights. Furthermore, repeated performance scenarios should be investigated to understand how IWU influences performance sustainability over multiple sprints. Finally, mechanistic explanations, such as the role of the respiratory metaboreflex, should be explored to better elucidate how IWU affects sprint performance.

## Supplementary Information

Below is the link to the electronic supplementary material.

Supplementary Material 1

## Data Availability

The datasets generated during and/or analysed during the current study are available in the fligshare repository, https://figshare.com/s/9e9886da77bff116c415?file=53623961.
