# Supplementary material for: Inspiratory muscle warm up improves 400 m performance in elite male runners
Source: Sci Rep. 2025 Aug 7;15:28879. doi: 10.1038/s41598-025-14797-0 (PMC12332055; doi:10.1038/s41598-025-14797-0)
Supplement: Supplementary file 1 — Supplementary Material 1 [file 41598_2025_14797_MOESM1_ESM.docx]

**Supplementary Materials**

**Tables**

**Table S1. The Changes in respiration levels in time flow as based on the warm-up protocols**

| **Variables** | **Warm up Protocol** | **^1^At rest** | **%** | **^2^After warm up** | **%** | **^3^Immediately**  **after run** | **%** | **^4^1ˈ after run** | **%** | **^5^3ˈ minute** | **%** | **^6^5ˈ minute** | **F** | **p** | **η2** |
| --- | --- | --- | --- | --- | --- | --- | --- | --- | --- | --- | --- | --- | --- | --- | --- |
| **MIP**  **(cmH_2_O)** | **^1^AW** | 138.84±5.87 | 1.21 | 140.53±6.19 | -15.81 | 118.30±5.08 | 1.63 | 120.23±5.23 | 3.51 | 124.46±3.99 | 1.42 | 126.23±4.72 | 117,128 | 0.001 | .937 |
|  | **p** | **AE: 1-2^x^** |  | **AE: 2-1^x^** |  | **all** |  | **all** |  | **AE: 5-6^x^** |  | **AE: 6-5^x^** |  |  |  |
|  | **^2^IWU_60_** | 139,53±6,72 | 7.71 | 150,30±6,95 | -11.41 | 133.15±5.72 | 3.06 | 137.23±5.27 | 1.50 | 139.30±5.76 | 0.99 | 140.69±6.07 | 167.882 | 0.001 | .933 |
|  | **p** | **AE: 1-4^x^** |  | **All** |  | **All** |  | **AE: 4-1^x^** |  | **all** |  | **all** |  |  |  |
|  | **^3^ IWU_40_** | 140.15±6.22 | 6.80 | 149.69±6.93 | -11.39 | 132.56±5.45 | 2.90 | 136.46±5.41 | 2.81 | 140.30±6.30 | 0.66 | 141.23±7.40 | 111.422 | 0.001 | .903 |
|  | **p** | **AE: 1-5^x^,1-6^x^** |  | **all** |  | **all** |  | **all** |  | **AE: 5-1^x^,5-6^x^** |  | **AE: 6-1^x^,6-5^x^** |  |  |  |
|  | **^4^SHAM** | 140.00±6.24 | 1.80 | 142.52±7.13 | -15.97 | 119.76±7.55 | 1.73 | 121.84±7.96 | 2.01 | 124.30±7.68 | 1.30 | 125.92±8.56 | 255.081 | 0.001 | .955 |
|  | **p** | **all** |  | **all** |  | **all** |  | **all** |  | **all** |  | **all** |  |  |  |
| ****Post hoc*** |  | **-** |  | ***1-2(6.95), 1-3(6.51),**  **2-4(5.17),**  **4-3(5.03)** |  | ***1-2(12.55), 1-3(12.09), 2-4 (10,05), 4-3(9,69)** |  | ***1-2(14,13), 1-3(13,49), 2-4(11,21), 4-3(10,71)** |  | ***1-2(11.92),**  **1-3(12.72),**  **2-4(-10.76),**  **4-3(12.87)** |  | ***1-2(11.45),**  **1-3(11.88),**  **2-4(-10.49),**  **4-3(12.15)** |  |  |  |
| **MEP**  **(cmH_2_O)** | **^1^AW** | 174.76±13.37 | 1.01 | 176.53±13.47 | -16.81 | 146.84±5.39 | 0.46 | 147.53±5.98 | 2.29 | 150.92±6.42 | 0.76 | 152.07±6.75 | 40.727 | 0.001 | .772 |
|  | **p** | **all** |  | **all** |  | **AE: 3-4^x^** |  | **AE: 4-3^x^** |  | **AE: 5-6^x^** |  | **AE: 6-5^x^** |  |  |  |
|  | **^2^IWU_60_** | 174.38±13.74 | 4.06 | 181.46±14.23 | -15.68 | 153.00±8.54 | 0.75 | 154.15±8.34 | 1.89 | 157.07±8.11 | 0.83 | 158.38±8.35 | 96.159 | 0.001 | .889 |
|  | **p** | **all** |  | **all** |  | **all** |  | **all** |  | **AE: 5-6^x^** |  | **AE: 6-5^x^** |  |  |  |
|  | **^3^ IWU_40_** | 173.69±13.66 | 3.98 | 180.61±14.81 | -12.13 | 158.69±12.02 | 2.08 | 162.00±11.96 | 3.17 | 167.15±13.01 | 3.58 | 173.15±13.12 | 175.345 | 0.001 | .936 |
|  | **p** | **AE: 1-6^x^** |  | **all** |  | **all** |  | **all** |  | **all** |  | **AE: 6-1^x^** |  |  |  |
|  | **^4^SHAM** | 174.46±13.64 | 1.80 | 177.61±13.21 | -16.84 | 147.69±5.64 | 0.83 | 148.92±5.86 | 0.98 | 150.38±6.19 | 1.07 | 152.00±6.32 | 40.883 | 0.001 | .773 |
|  | **p** | **all** |  | **all** |  | **all** |  | **AE: 4-5^x^** |  | **AE: 5-4^x^** |  | **all** |  |  |  |
| ***Post hoc** |  | **-** |  | **-** |  | **1-3(8.07),**  **3-4(-6.93)** |  | **1-3(9.80),**  **2-3(5.09),**  **3-4(-8.07)** |  | **1-3(10.75),**  **2-3(6.41),**  **3-4(-10.03)** |  | **1-3(13.86),**  **2-3(9.32),**  **3-4(-12.21)** |  |  |  |
| **PIFR**  **(L*^s-1^)** | **^1^AW** | 6.20±0.55 | 1.61 | 6.30±0.53 | -15.87 | 5.30±0.43 | 0.56 | 5.33±0.45 | 1.50 | 5.41±0.45 | 1.10 | 5.47±0.44 | 82.738 | 0.001 | .873 |
|  | **p** | **all** |  | **All** |  | **AE: 3-4^x^,3-5 ^x^, 3-6 ^x^** |  | **AE: 4-3^x^,4-5 ^x^** |  | **AE: 5-3^x^,5-4^x^,5-6^x^** |  | **AE: 6-3^x^,6-5^x^** |  |  |  |
|  | **^2^IWU_60_** | 6.18±0.54 | 2.42 | 6.33±0.55 | -11.05 | 5.63±0.55 | 0.71 | 5.67±0.60 | 1.76 | 5.77±0.55 | 1.21 | 5.84±0.55 | 50.272 | 0.001 | .807 |
|  | **p** | **all** |  | **all** |  | **AE: 3-4^x^** |  | **AE: 4-3^x^** |  | **all** |  | **all** |  |  |  |
|  | **^3^ IWU_40_** | 6.16±0.53 | 2.43 | 6.31±0.55 | -12.36 | 5.53±0.48 | 2.35 | 5.66±0.51 | 3.00 | 5.83±0.54 | 2.22 | 5.96±0.55 | 74.075 | 0.001 | .861 |
|  | **p** | **AE: 1-6^x^** |  | **all** |  | **all** |  | **all** |  | **all** |  | **AE: 6-1^x^,** |  |  |  |
|  | **^4^SHAM** | 6.23±0.51 | 0.80 | 6.28±0.53 | -15.44 | 5.31±0.45 | 0.18 | 5.32±0.46 | 1.31 | 5.39±0.46 | 1.11 | 5.45±0.46 | 99.372 | 0.001 | .892 |
|  | **p** | **AE: 1-2^x^** |  | **AE: 2-1^x^** |  | **AE: 3-4^x^,3-5 ^x^, 3-6 ^x^** |  | **AE: 4-3^x^,4-5 ^x^,4-6^x^** |  | **AE: 5-3^x^,5-4^x^,5-6^x^** |  | **AE: 6-3^x^, 6-4^x^,6-5^x^** |  |  |  |
| ***Post hoc** |  | **-** |  | **-** |  | **-** |  | **-** |  | **-** |  | **-** |  |  |  |
| **IV**  **(L)** | **^1^AW** | 3.91±0.14 | 0.51 | 3.93±0.13 | -10.94 | 3.50±0.15 | 0.00 | 3.50±0.16 | 2.85 | 3.60±0.18 | 0.55 | 3.62±0.17 | 98.489 | 0.001 | .891 |
|  | **p** | **AE: 1-2^x^** |  | **AE: 2-1^x^** |  | **AE: 3-4^x^** |  | **AE: 4-3^x^** |  | **AE: 5-6^x^** |  | **AE: 6-5^x^** |  |  |  |
|  | **^2^IWU_60_** | 3.83±0.30 | 3.39 | 3.96±0.27 | -7.32 | 3.67±0.25 | 0.81 | 3.70±0.28 | 1.08 | 3.74±0.30 | 0.53 | 3.76±0.32 | 33.405 | 0.001 | .736 |
|  | **p** | **AE: 1-5^x^, 1-6^x^** |  | **All** |  | **AE: 3-4^x^,3-5 ^x^, 3-6 ^x^** |  | **AE: 4-3^x^,4-5 ^x^,4-6^x^** |  | **AE: 5-1^x^ 5-3^x^,5-4^x^,5-6^x^** |  | **AE: 6-1^x^ 6-3^x^, 6-4^x^,6-5^x^** |  |  |  |
|  | **^3^ IWU_40_** | 3.86±0.25 | 1.81 | 3.93±0.23 | -7.63 | 3.63±0.21 | 1.65 | 3.69±0.23 | 2.98 | 3.80±0.23 | 1.05 | 3.84±0.25 | 46.337 | 0.001 | .794 |
|  | **p** | **AE: 1-2^x,^ 1-5^x^,1-6^x^** |  | **AE: 2-1^x^ , 2-6^x^** |  | **all** |  | **all** |  | **AE: 5-1^x^,5-6^x^** |  | **AE: 6-1^x^ 6-2^x^, 6-5^x^** |  |  |  |
|  | **^4^SHAM** | 3.87±0.20 | 0.25 | 3.88±0.18 | -9.53 | 3.51±0.13 | 0.28 | 3.52±0.14 | 1.42 | 3.57±0.18 | 0.84 | 3.60±0.18 | 54.494 | 0.001 | .821 |
|  | **p** | **AE: 1-2^x^** |  | **AE: 2-1^x^** |  | **AE: 3-4^x^,3-5 ^x^, 3-6 ^x^** |  | **AE: 4-3^x^,4-5 ^x^,4-6^x^** |  | **AE: 5-3^x^,5-4^x^,5-6^x^** |  | **AE: 6-3^x^, 6-4^x^,6-5^x^** |  |  |  |
| ***Post hoc** |  | **-** |  | **-** |  | **-** |  | **-** |  | **-** |  | **-** |  | | |

Values are mean and standard deviation. MEP: maximum expiratory pressure; MIP: maximum inspiratory pressure; PIFR: peak inspiratory flow rate; IV: inhaled volume.

all: all of variables were significant with each other (p=.001)

AE: all of variables were significant except “x”

**Table S2. The Changes in lactate concentrations in time flow as based on the warm-up protocols**

| **Variables** | **Warm up Protocols** | **^1^Rest** | **^%^** | **^2^After warm up** | **^%^** | **^3^Immediately**  **after run** | **^%^** | **^4^1ˈ after run** | **^%^** | **^5^3ˈ after run** | **^%^** | **^6^5ˈ after run** | **F** | **p** | **η2** |
| --- | --- | --- | --- | --- | --- | --- | --- | --- | --- | --- | --- | --- | --- | --- | --- |
| **Blood Lactate**  **(mmol*L^-1^)** | **^1^AW** | 0.92±0.12 | 292.39 | 3.61±0.26 | 403.32 | 18.17±0.35 | 0.99 | 18.35±0.43 | 1.03 | 18.54±0.61 | -15.26 | 15.71±0.52 | 8359.268 | 0.001 | .999 |
|  | **p** | **all** |  | **all** |  | **all** |  | **AE: 4-5^x^** |  | **AE: 5-4^x^** |  | **all** |  |  |  |
|  | **^2^IWU_60_** | 0.88±0.34 | 369.31 | 4.13±0.19 | 327.36 | 17.65±0.35 | -1.69 | 17.35±0.28 | -0.74 | 17.22±0.26 | -17.18 | 14.26±0.35 | 10505.093 | 0.001 | .999 |
|  | **p** | **all** |  | **all** |  | **all** |  | **all** |  | **all** |  | **all** |  |  |  |
|  | **^3^ IWU_40_** | 0.96±0.23 | 320.83 | 4.04±0.17 | 337.37 | 17.67±0.40 | -1.58 | 17.39±0.40 | -1.09 | 17.20±0.41 | -18.43 | 14.03±0.46 | 9955.094 | 0.001 | .999 |
|  | **p** | **all** |  | **all** |  | **all** |  | **all** |  | **all** |  | **all** |  |  |  |
|  | **^4^SHAM** | 0.96±0.17 | 294.79 | 3.79±0.28 | 378.62 | 18.14±0.37 | 0.55 | 18.24±0.48 | 0.32 | 18.30±0.46 | -15.57 | 15.45±0.33 | 10646.272 | 0.001 | .999 |
|  | **p** | **all** |  | **all** |  | **AE: 3-4^x^. 3-5^x^** |  | **AE: 4-3^x^. 4-5^x^** |  | **AE: 5-3^x^. 5-4^x^** |  | **all** |  |  |  |
| ***Post hoc** |  |  |  | ***1-2(14.40)**  **1-3(11.91)**  **2-4(-8.23)**  **4-3(6.59)** |  | ***1-2(-2.86)**  **1-3(-2.75)**  **2-4(2.77)**  **4-3(-2.59)** |  | ***1-2(-5.44)**  **1-3(-5.23)**  **2-4(5.12)**  **4-3(-4.66)** |  | ***1-2(-7.11)**  **1-3(-7.22)**  **2-4(6.27)**  **4-3(-6.01)** |  | ***1-2(-9.22)**  **1-3(-10.69)**  **2-4(8.34)**  **4-3(-9.19)** |  | | |

all: all of variables were significant with each other (p=.001)

AE: all of variables were significant except “x”

**Table S3. The Changes in the heart rates in time flow as based on the warm up protocols**

| **Variables** | **Warm up Protocol** | **^2^After warm up** | **^%^** | **^3^Immediately**  **after run** | **^%^** | **^4^1ˈ after run** | **^%^** | **^5^3ˈ after run** | **^%^** | **^6^5ˈ after run** | **F** | **p** | **η2** |
| --- | --- | --- | --- | --- | --- | --- | --- | --- | --- | --- | --- | --- | --- |
| **Heart rate**  **(Bpm)** | **^1^AW** | 102.15±3.28 | 80.04 | 183.92±3.94 | -13.29 | 159.46±3.92 | -27.78 | 115.15±6.06 | -8.41 | 105.46±4.79 | 2161.663 | .000 | .994 |
|  | **p** | **AE: 1-5^x^** |  | **all** |  | **all** |  | **all** |  | **AE: 5-1^x^** |  |  |  |
|  | **^2^IWU_60_** | 103.30±2.17 | 77.82 | 183.69±3.63 | -15.2 | 155.76±5.46 | - 29.03 | 110.53±5.02 | -6.12 | 103.76±5.03 | 1587.528 | .000 | .992 |
|  | **p** | **AE: 1-5^x^** |  | **all** |  | **all** |  | **all** |  | **AE: 5-1^x^** |  |  |  |
|  | **^3^ IWU_40_** | 104.23±3.83 | 74.75 | 182.15±4.29 | -15.87 | 153.23±4.93 | -29.47 | 108.07±4.05 | -5.55 | 102.07±5.54 | 1462.694 | .000 | .992 |
|  | **p** | **AE: 1-4^x^. 1-5^x^** |  | **all** |  | **all** |  | **AE: 4-1^x^** |  | **AE: 5-1^x^** |  |  |  |
|  | **^4^SHAM** | 103.46±3.52 | 77.69 | 183.84±4.25 | -13.38 | 159.23±3.74 | -28.16 | 114.38±5.47 | -9.34 | 103.69±4.28 | 1840.161 | .000 | .994 |
|  | **p** | **AE: 1-5^x^** |  | **all** |  | **all** |  | **all** |  | **AE: 5-1^x^** |  |  |  |
| ***Post Hoc** |  | **-** |  | **-** |  | **1-3(-3.90)**  **3-4(3.91)** |  | **1-3(-6.14)**  **3-4(5.83)** |  | **-** |  | | |

all: all of variables were significant with each other (p=.001)

AE: all of variables were significant except “x”
